# Differential Levels of Cecal Colonization by *Salmonella* Enteritidis in Chickens Triggers Distinct Immune Kinome Profiles

**DOI:** 10.3389/fvets.2017.00214

**Published:** 2017-12-13

**Authors:** Christina L. Swaggerty, Michael H. Kogut, Haiqi He, Kenneth J. Genovese, Casey Johnson, Ryan J. Arsenault

**Affiliations:** ^1^U.S. Department of Agriculture, Agricultural Research Service, College Station, TX, United States; ^2^Department of Animal and Food Sciences, University of Delaware, Newark, DE, United States

**Keywords:** chicken, kinome, peptide array, resistance, *Salmonella*

## Abstract

*Salmonella enterica* serovar Enteritidis are facultative intracellular bacteria that cause disease in numerous species. *Salmonella*-related infections originating from poultry and/or poultry products are a major cause of human foodborne illness with *S*. Enteritidis the leading cause worldwide. Despite the importance of *Salmonella* to human health and chickens being a reservoir, little is known of the response to infection within the chicken gastrointestinal tract. Using chicken-specific kinome immune peptide arrays we compared a detailed kinomic analysis of the chicken jejunal immune response in a single line of birds with high and low *Salmonella* loads. Four-day-old chicks were challenged with *S*. Enteritidis (10^5^ cfu) and cecal content and a section of jejunum collected at three times: early [4–7 days post-infection (dpi)], middle (10–17 dpi), and late (24–37 dpi). *Salmonella* colonization was enumerated and birds with the highest (*n* = 4) and lowest (*n* = 4) loads at each time were selected for kinomic analyses. Key biological processes associated with lower loads of *Salmonella* clustered around immune responses, including cell surface receptor signaling pathway, positive regulation of cellular processes, defense response, innate immune response, regulation of immune response, immune system process, and regulation of signaling. Further evaluation showed specific pathways including chemokine, Jak–Stat, mitogen activated protein kinase, and T cell receptor signaling pathways were also associated with increased resistance. Collectively, these findings demonstrate that it is possible to identify key mechanisms and pathways that are associated with increased resistance against *S*. Enteritidis cecal colonization in chickens. Therefore, providing a foundation for future studies to identify specific proteins within these pathways that are associated with resistance, which could provide breeders additional biomarkers to identify birds naturally more resistant to this important foodborne pathogen.

## Introduction

*Salmonella enterica* serovar Enteritidis (*S*. Enteritidis) is the leading cause of bacterial-derived foodborne illness worldwide (1), and *Salmonella*-related infections originating from poultry and/or poultry products are a significant cause of these human illnesses (2). Studies of global gene expression are informative, but many cellular processes are regulated independently of changes in transcription or translation through post-translational modifications of host proteins.

Phosphorylation is the predominant mechanism of post-translational modification for regulation of protein function and has a central role in virtually every cellular event, as well as strong linkages with many diseases (3). Protein kinases are essential components of all cell signaling networks and events and, therefore, regulate fundamental biological processes (BPs) ranging from cellular growth to death and all processes in between (4). Examining the active kinase enzymes responsible for these phosphorylation events can provide key information into numerous host and cellular functions; therefore, there is a considerable interest in defining kinase activities. Active peptides that represent target sites of kinase enzymes can be printed onto array surfaces/slides (5), and are emerging as an important means of characterizing kinome activity (6). Global analysis of the kinome provides information on the abundance, activity, substrate specificity, phosphorylation pattern, and mutational status (4). Our laboratory has designed and developed chicken-specific arrays targeting immune and metabolism kinome activities (7, 8). Kinome analysis using peptide arrays provide site-specific information, display similar biochemical properties to the full protein, and have demonstrated considerable potential as a cost-effective, high-throughput approach for defining phosphorylation-mediated events (9); therefore, potentially making it possible to identify specific biomarkers associated with a desired phenotype.

Previously, our laboratory developed a novel selection method based on identification and selection of chickens with naturally high levels of pro-inflammatory mediators, including interleukin (IL) 6, CXCLi2, and CCLi2 and demonstrated the resultant chickens are more resistant to the foodborne pathogen *S*. Enteritidis (10) and other key foodborne and poultry pathogens (11–13). While our original selection strategy proved effective, an approach utilizing kinome analysis could provide a new molecular-based tool that offers the potential for high-throughput screening and selection of chickens. Identification of specific biomarkers that the poultry industry could use to select individual birds that are more resistant to cecal colonization with *S*. Enteritidis would be beneficial to the industry. This could potentially lead to either fewer *S*. Enteritidis positive birds entering the processing plant or reducing the load of bacteria the birds are carrying and, therefore, fewer positive chicken products reaching the consumer.

Within a single genetic line of birds, one would expect to find individuals that are more or less susceptible to *Salmonella* than some flock mates. The objectives of this study were to (1) identify chickens within a single genetic population with high and low loads of *S*. Enteritidis cecal colonization following an oral challenge, (2) perform innate immune kinome analysis to monitor kinase-mediated signaling activity on jejunal samples from non-challenged, high load *S*. Enteritidis, and low load *S*. Enteritidis birds at three distinct time points, and (3) identify the immunological processes and signaling pathways associated with enhanced resistance to *S*. Enteritidis cecal colonization within a single line of chickens at three times over the 42-day grow-out.

## Materials and Methods

### Experimental Animals

All experiments were conducted according to guidelines established by the United States Department of Agriculture (USDA) animal care and use committee, which operates in accordance with established principles (14). Broiler chickens from a single genetic lineage were obtained from a commercial hatchery. At hatch, straight-run (mixed sexes) chicks were placed in floor pens (4 m × 4 m) containing wood shavings, supplemental heat, water and a balanced, un-medicated corn, and soybean meal-based chick starter diet *ad libitum*. The feed contained 23% protein and 3,200 kcal of metabolizable energy/kg of diet, and all other nutrient levels met or exceeded established requirements (15). The birds were not vaccinated or given any medications during the course of the study.

### Bacteria Preparation

A poultry isolate of *Salmonella enterica* serovar Enteritidis (*S*. Enteritidis) was obtained from the National Veterinary Services Laboratory (Ames, IA, USA), and was selected for resistance to nalidixic acid and novobiocin and maintained in tryptic soy broth (Difco Laboratories, Sparks, MD, USA) containing antibiotics (20 µg/mL nalidixic acid and 25 µg/mL novobiocin; Sigma Chemical Co., St. Louis, MO, USA). A stock culture was prepared in sterile phosphate buffered saline (PBS) and adjusted to a concentration of 1 × 10^9^ colony forming units (cfu)/mL as previously described (16). The challenges were then diluted from the 1 × 10^9^ cfu/mL stock culture to the desired concentration. The viable cell concentration of the challenge dose for each experiment was determined by colony counts on XLT4 agar base plates with XLT4 supplement (Difco) and nalidixic acid and novobiocin (XLT-NN).

### Bacterial Challenge and Recovery

Four-day-old broiler chicks were challenged orally with *S*. Enteritidis (0.5 mL; 4.8 × 10^5^ cfu/chick) while controls were administered sterile PBS; 0.5 mL. Cloacal swabs were collected 3 days post challenge to confirm the controls were not infected and that all birds that were challenged were culture positive for *S*. Enteritidis. Briefly, a sterile cotton swab was gently inserted into the cloaca and a fecal sample was collected. The entire swab and sample for each bird was then placed into a separate tube containing tetrathionate enrichment broth (10 mL, Difco) and incubated overnight at 41°C. Following enrichment, 10 µL were streaked onto XLT-NN plates, incubated 24 h at 41°C, then the plates examined for non-lactose fermenting NN-resistant *Salmonella* colonies. Representative colonies were confirmed positive by plate agglutination using specific Group D_1_ antisera (Difco).

### Sample Collection and Processing

One-day-old broiler chickens were randomly distributed into two experimental groups: non-infected control and infected (*n* = 50). Early samples were collected between 4 and 7 days post-infection (dpi), middle samples were collected between 10 and 17 dpi, and late samples were collected between 24 and 37 dpi. The experiments were conducted on two separate occasions.

Control and infected chickens (*n* = 10) were euthanized by cervical dislocation and necropsied at three timeframes (early, middle, and late) over the course of a 42-day grow-out. The cecum from each chicken was removed aseptically, and the contents (0.25 g) were serially diluted to 1:100, 1:1,000, or 1:10,000 and spread onto XLT-NN plates to enumerate *S*. Enteritidis. The plates were incubated at 41°C for 24 h, and the number of NN-resistant *S*. Enteritidis cells per g of cecal contents determined. A piece of jejunum (100 mg) was collected and rinsed with PBS to remove content and then placed into a cryovial containing 1.5 mL RNA*later* RNA stabilization reagent (Qiagen, Valencia, CA, USA) and stored at −20°C until tissue homogenization and RNA isolation was performed for quantitative real-time Reverse Transcriptase-PCR (qRT-PCR). Additionally, a section of jejunum (100 mg) was collected from each bird, rinsed with PBS to remove content, and then immediately flash frozen in liquid nitrogen to preserve kinase enzymatic activity for the array. Samples were taken from liquid nitrogen and transferred to a −80°C freezer until further experimental procedures were conducted. Following microbiological analysis of the cecal contents (described previously), the jejunum tissues from four birds with the highest and four birds with the lowest levels of recoverable *S*. Enteritidis (out of the 10 birds per time) were used for the peptide arrays.

### Kinome (Peptide) Array

PepStar peptide microarrays were obtained from JPT Peptide Technologies GmbH (Berlin, Germany), and the peptide array protocol was carried out as previously described (6) with the following modifications (8, 17). Jejunum tissue samples were weighed to obtain a consistent 40 mg sample for the array protocol. Samples were homogenized by a hand-held TissueRuptor (Qiagen, Valencia, CA, USA) in 100 µL of lysis buffer (20 mM Tris–HCl pH 7.5, 150 mM NaCl, 1 mM Ethylenediaminetetraacetic acid, 1 mM ethylene glycol tetraacetic acid, 1% Triton X-100, 2.5 mM sodium pyrophosphate, 1 mM Na_3_VO_4_, 1 mM NaF, 1 µg/mL leupeptin, 1 g/mL aprotinin, and 1 mM Phenylmethylsulphonyl fluoride). All chemicals purchased from Sigma-Aldrich, Co. (St. Louis, MO, USA) unless otherwise indicated.

### Antibody Array

The Phospho Explorer Antibody Array kit (catalog PEX100; Full Moon BioSystems, Sunnyvale, CA, USA) consists of over 1,300 antibodies from over 30 signaling pathways and is an alternative approach to procuring phosphor-specific antibodies individually and performing numerous western blot assays. The protocol was carried out as per manufacturer’s instructions with the exception that the tissue was homogenized using a hand-held TissueRuptor (Qiagen, Valencia, CA, USA) instead of the bead and vortex method suggested in the kit.

### Data Analysis: Kinome and Antibody Arrays

Data normalization and analysis was performed for both the kinome and antibody microarrays as described (17). Images were gridded using GenePix Pro software, and the spot intensity signal was collected as the mean of pixel intensity using local feature background intensity calculation with the default scanner saturation level. The data was then analyzed using the Platform for Intelligent Integrated Kinome Analysis (PIIKA2) peptide array analysis software (http://saphire.usask.ca/saphire/piika/index.html). Briefly, the resulting data points were normalized to eliminate variance due to technical variation, for example, random variation in staining intensity between arrays or between array blocks within an array. Variance stabilization and normalization was performed. Note: as the arrays were printed with triplicate peptide blocks there are three values for each peptide. Using the normalized data set comparisons between treatment and control groups were performed, calculating fold-change and a significance *P*-value. The *P*-value is calculated by conducting a one-sided paired *t*-test between treatment and control values for a given peptide.

This consistent analysis method facilitates a more direct comparison between the two distinct array datasets and allows for a statistically robust analysis of the phosphorylation events being measured. Gene ontology (GO) and Kyoto Encyclopedia of Genes and Genomes (KEGG) pathway analysis was performed by uploading the statistically significant peptide lists to the Search Tool for the Retrieval of Interacting Genes (STRING^1^) (18).

### Isolation of Total RNA for qRT-PCR

Tissue homogenization was performed using a BeadBug microtube homogenizer (Benchmark Scientific, Edison, NJ, USA). Briefly, a piece of tissue (30–40 mg) was removed from RNA*later* and placed in a 2 mL prefilled tube containing 1.5 mm high impact zirconium beads (TriplePure M-Bio Grade; Benchmark Scientific). Lysis buffer (350 µL; RNeasy Mini Kit; Qiagen) was added and the sample was homogenized in the BeadBug for 2 min on the maximum speed. Total RNA was then isolated from the homogenized samples according to the manufacturer’s instructions, eluted with 50 µL RNase-free water, and stored at −80°C until qRT-PCR analyses performed.

### Quantitative Real-time RT-PCR

Interleukin 6 and CXCLi2 mRNA expression was quantified using a well-described method. Primers and probes for cytokines, chemokines, and 28S RNA-specific amplification has been previously described (19–21). The qRT-PCR was performed using the TaqMan one-step RT-PCR master mix reagents (Applied Biosystems, Branchburg, NJ). Amplification and detection of specific products were performed using the Applied Biosystems 7500 Fast Real-Time PCR System with the following cycle profile: one cycle of 48°C for 30 min, 95°C for 20 s, and 40 cycles of 95°C for 3 s and 60°C for 30 s. Quantification was based on the increased fluorescence detected by the 7500 Fast Sequence Detection System due to hydrolysis of the target-specific probes by the 5′ nuclease activity of the *rTth* DNA polymerase during PCR amplification. To correct for differences in RNA levels between samples within the experiment, the correction factor for each sample was calculated by dividing the mean threshold cycle (*C_t_*) value for 28S rRNA-specific product for each sample, by the overall mean *C_t_* value for the 28S rRNA-specific product from all samples. The corrected cytokine mean is calculated: (Average of each replicate × cytokine slope)/28s slope × 28 s correction factor. The data shown are corrected 40-*C*_t_ values.

### Statistical Analyses

The mean and SEM for each cytokine/chemokine were calculated at each time and statistical analyses performed (Student’s *t*-test); comparisons were made between a single timeframe comparing the birds with high and low levels of *S*. Enteritidis cecal colonization. No statistical analysis was performed for the differences in bacterial load. Details for the array analysis are provided in the Data Analysis: Kinome and Antibody Arrays section described above. For all analyses, significance was considered if *P* ≤ 0.05.

## Results

### *S*. Enteritidis Colonization

Cloacal swabs were collected on all birds in the study three days post challenge to confirm the controls were not infected and that all challenged birds were culture positive for the challenge strain of *S*. Enteritidis. All birds administered the challenge were positive for *S*. Enteritidis while all of the controls were negative (data not shown).

**Figure 1:**
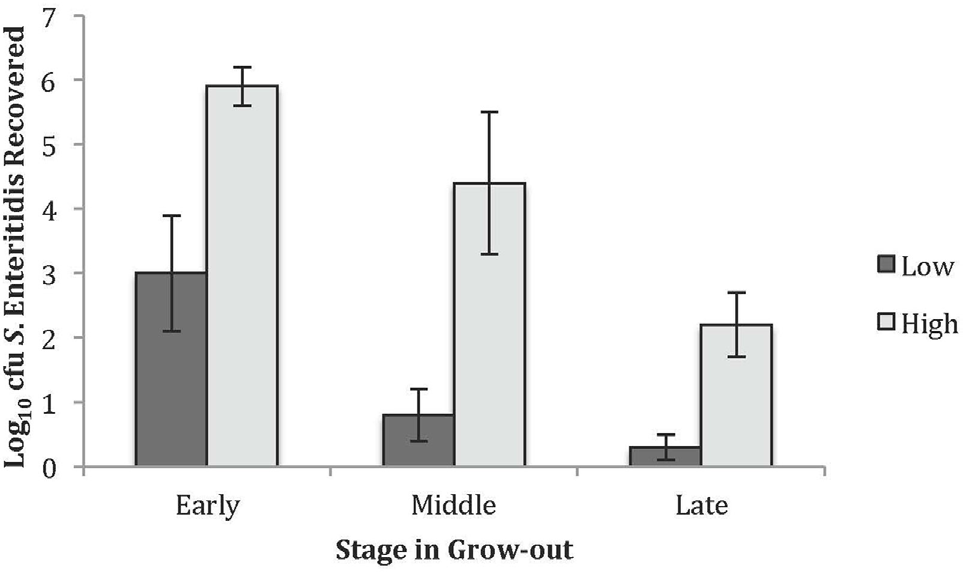
Recovered *Salmonella* Enteritidis in the high and low groups used for the peptide and antibody arrays. The average Log_10_ colony forming units recovered for the low and high *S*. Enteritidis groups at the early, middle, and late times.

Within this single line of birds, varying levels of *S*. Enteritidis cecal colonization were observed and, therefore, studying birds with relatively high and low numbers of recoverable *S*. Enteritidis was pursued. The levels of *S*. Enteritidis colonization for the high and low groups at each timeframe are summarized in Figure 1. Chickens in the early time point and designated to the low group had 3.0 ± 0.9 Log_10_ cfu of recoverable *S*. Enteritidis while the birds in the high group had 5.9 ± 0.3 Log_10_ cfu. The values for the middle group were 0.8 ± 0.4 and 4.4 ± 1.1 Log_10_ cfu *S*. Enteritidis for the low and high groups, respectively. The recoverable *S*. Enteritidis continued to decline by the late timeframe and were 0.3 ± 0.2 and 2.2 ± 0.5 Log_10_ cfu *S*. Enteritidis for the low and high groups, respectively.

### GO BPs

The GO Consortium assigns defined terms representing gene product properties and is broken into three categories: cellular component, molecular function, and BP. The BP terms include operations or sets of molecular events with a defined beginning and end that pertain to the functioning of the integrated living units.^2^ Using STRING functionality, GO results for BP were generated for each dataset. The total number of BPs associated with each time and bacterial load were: early low = 1,218; early high = 617; middle low = 1,084; middle high = 1,001; late low = 942; and late high = 1,049 (data not shown). The most significant (based on *P*-value) immunologically relevant GO BP were selected for further analysis. Analysis of the kinome data showed distinct differences in the observed BP between the loads of bacteria, and some of the central differences are provided in Table 1. Each term listed had a false discovery rate (FDR) *P* ≤ 0.01. The BP with high numbers of peptide phosphorylation events associated with low levels of *S*. Enteritidis colonization regardless of time (early, middle, or late) are clustered around immune responses and subsequent signaling pathways and include: cell surface receptor signaling pathway, positive regulation of cellular processes, defense response, innate immune response, regulation of immune response, immune system process, and regulation of signaling. Activation of the BP were observed at the early, middle, and late infection times, but the numbers of differentially phosphorylated peptides between the low/high loads of *S*. Enteritidis were greatest at the early and middle points. By late in the infection, the numbers of peptides was similar between the birds with low and high loads of *S*. Enteritidis as might be expected based on the overall lower numbers of bacteria recovered (Figure 1). These data point to the early immune mechanisms that aid in controlling *S*. Enteritidis.

**Table 1:** Gene ontology (GO) biological process (BPs) terms identified using the peptide array and the number of differentially phosphorylated peptides associated with high and low loads of *Salmonella* Enteritidis colonization.

GO ID	BP term	Early low SE	Early high SE	Middle low SE	Middle high SE	Late low SE	Late high SE
GO.0050776	Regulation of immune response	60	26	52	40	41	43
GO.0045087	Innate immune response	61	31	55	42	44	43
GO.0002764	Immune response-regulating signaling pathway	48	21	45	34	34	35
GO.0002768	Immune response-regulating cell surface receptor signaling pathway	40	17	38	30	31	28
GO.0002684	Positive regulation of immune system process	45	21	45	29	32	29
GO.0002376	Immune system process	60	28	63	47	46	42
GO.0034097	Response to cytokine	37	16	26	15	24	22
GO.0043549	Regulation of kinase activity	38	17	40	33	25	28
GO.0034142	TLR4 signaling pathway	18	7	17	11	10	9
GO.0000165	Mitogen activated protein kinase cascade	20	8	19	21	12	12
GO.0009617	Response to bacterium	23	6	16	11	14	18
GO.0048522	Positive regulation of cellular process	77	36	66	52	49	54
GO.0006935	Chemotaxis	27	12	22	22	19	7
GO.0006909	Phagocytosis	16	6	11	8	12	12
GO.0006954	Inflammatory response	17	6	9	10	11	11
GO.0001932	Regulation of protein phosphorylation	49	22	48	38	29	37
GO.0006952	Defense response	67	30	58	43	47	43
GO.0007166	Cell surface receptor signaling pathway	81	42	72	58	57	60

*SE, S. Enteritidis*.

### KEGG Pathway Activation

Using STRING functionality, KEGG pathway results were generated for each dataset. To ensure that changes in phosphorylation were a direct result of the infection, the results were corrected using the appropriate age-matched controls. The KEGG pathway results showed numerous pathways that were significantly different between the birds with high and low loads of *S*. Enteritidis at each time of the infection (*P* ≤ 0.05 FDR). In order to be included, a pathway had to be significant for each bird within a group and time. Pathways that were not significant for each bird at a specific time and bacterial load were excluded. Additionally, the numbers for a subset of the significantly different peptides within each of the KEGG pathways are also provided. The numbers shown are a small fraction of the total number of significant peptides within a specific pathway; however, as based on our criteria to be included in the dataset, a peptide had to be statistically significantly different from control for every bird in a given group (i.e., every early/low bird, early/high bird, middle/low bird, middle/high bird, late/low bird, or late/high birds).

The significant KEGG pathways observed at the early time are shown in Table 2, and the pathways for the middle and late times are provided in Tables 3 and 4, respectively. Pathways listed in bold showed statistically significant changes at all time points in birds with either low or high loads of *S*. Enteritidis. The common pathways observed in chickens with low loads of *S*. Enteritidis included: chemokine signaling pathway, Fc ε RI signaling pathway, focal adhesion, insulin signaling pathway, Jak–Stat signaling pathway, mitogen-activated protein kinase (MAPK) signaling pathway, neurotrophin signaling pathway, pathways in cancer, T cell receptor signaling pathway, and Tuberculosis. The specific proteins associated with each of the pathways, including those specifically related to cancer, would also be pivotal in determining the hosts’ immunological response against a challenge and/or disease. The only pathway that was significantly different at each time in birds with high levels of *S*. Enteritidis colonization was the MAPK signaling pathway.

**Table 2:** Kyoto Encyclopedia of Genes and Genomes (KEGG) pathways identified with the peptide array at the early stage infections in chickens with high and low levels of *Salmonella* Enteritidis colonization.

High *S*. Enteritidis	Number of peptides	Low *S*. Enteritidis	Number of peptides
B cell receptor signaling pathway	4	B cell receptor signaling pathway	4
Mitogen activated protein kinase **(MAPK) signaling pathway**	**6**	Chagas disease	4
		**Chemokine signaling pathway**	**4**
		Epithelial cell signaling pathway in *Helicobacter pylori* infection	2
		ErbB signaling pathway	6
		**Fc ε RI signaling pathway**	**5**
		Fc-γ receptor-mediated phagocytosis	5
		**Focal adhesion**	**6**
		GnRH signaling pathway	3
		**Insulin signaling pathway**	**7**
		**Jak–Stat signaling pathway**	**7**
		**MAPK signaling pathway**	**8**
		mTor signaling pathway	2
		Natural killer cell mediated cytotoxicity	6
		**Neurotrophin signaling pathway**	**6**
		Osteoclast differentiation	4
		**Pathways in cancer**	**10**
		**T cell receptor signaling pathway**	**6**
		Toll-like receptor signaling pathway	2
		Toxoplasmosis	3
		**Tuberculosis**	**3**
		VEGF signaling pathway	5

*Pathways listed in bold showed statistically significant changes at all time points in birds with either low or high loads of S. Enteritidis*.

**Table 3:** Kyoto Encyclopedia of Genes and Genomes (KEGG) pathways identified with the peptide array at the middle stage infections in chickens with high and low levels of *Salmonella* Enteritidis colonization.

High *S*. Enteritidis	Number of peptides	Low *S*. Enteritidis	Number of peptides
Bacterial invasion of epithelial cells	5	**Chemokine signaling pathway**	**5**
Chemokine signaling pathway	7	ErbB signaling pathway	4
Chronic myeloid leukemia	4	**Fc ε RI signaling pathway**	**2**
ErbB signaling pathway	7	**Focal adhesion**	**2**
Fc ε RI signaling pathway	1	GnRH signaling pathway	4
Focal adhesion	6	**Insulin signaling pathway**	**3**
Insulin signaling pathway	5	**Jak–Stat signaling pathway**	**4**
Mitogen activated protein kinase (**MAPK) signaling pathway**	**6**	Leukocyte transendothelial migration	2
Natural killer cell mediated cytotoxicity	4	**MAPK signaling pathway**	**4**
Neurotrophin signaling pathway	6	mTor signaling pathway	2
Pathways in cancer	6	**Neurotrophin signaling pathway**	**5**
T cell receptor signaling pathway	4	**Pathways in cancer**	**2**
		Regulation of actin cytoskeleton	1
		**T cell receptor signaling pathway**	**1**
		**Tuberculosis**	**3**

*Pathways listed in bold showed statistically significant changes at all time points in birds with either low or high loads of S. Enteritidis*.

**Table 4:** Kyoto Encyclopedia of Genes and Genomes (KEGG) pathways identified with the peptide array at the late stage infections in chickens with high and low levels of *Salmonella* Enteritidis colonization.

High *S*. Enteritidis	Number of peptides	Low *S*. Enteritidis	Number of peptides
Endocytosis	2	B cell receptor signaling pathway	3
ErbB signaling pathway	3	**Chemokine signaling pathway**	**5**
Focal adhesion	3	ErbB signaling pathway	3
Jak–Stat signaling pathway	2	**Fc ε RI signaling pathway**	**3**
mitogen activated protein kinase (**MAPK) signaling pathway**	**2**	Fc-γ R-mediated phagocytosis	2
Neurotrophin signaling pathway	2	**Focal adhesion**	**3**
Osteoclast differentiation	1	**Insulin signaling pathway**	**2**
Pathways in cancer	3	**Jak–Stat signaling pathway**	**3**
T cell receptor signaling pathway	1	**MAPK signaling pathway**	**1**
Toll-like receptor signaling pathway	2	Natural killer cell mediated cytotoxicity	2
Toxoplasmosis	2	**Neurotrophin signaling pathway**	**3**
Tuberculosis	3	Osteoclast differentiation	3
VEGF signaling pathway	2	**Pathways in cancer**	**5**
		**T cell receptor signaling pathway**	**2**
		Toll-like receptor signaling pathway	2
		Toxoplasmosis	2
		**Tuberculosis**	**2**
		VEGF signaling pathway	3

*Pathways listed in bold showed statistically significant changes at all time points in birds with either low or high loads of S. Enteritidis*.

**Figure 2:**
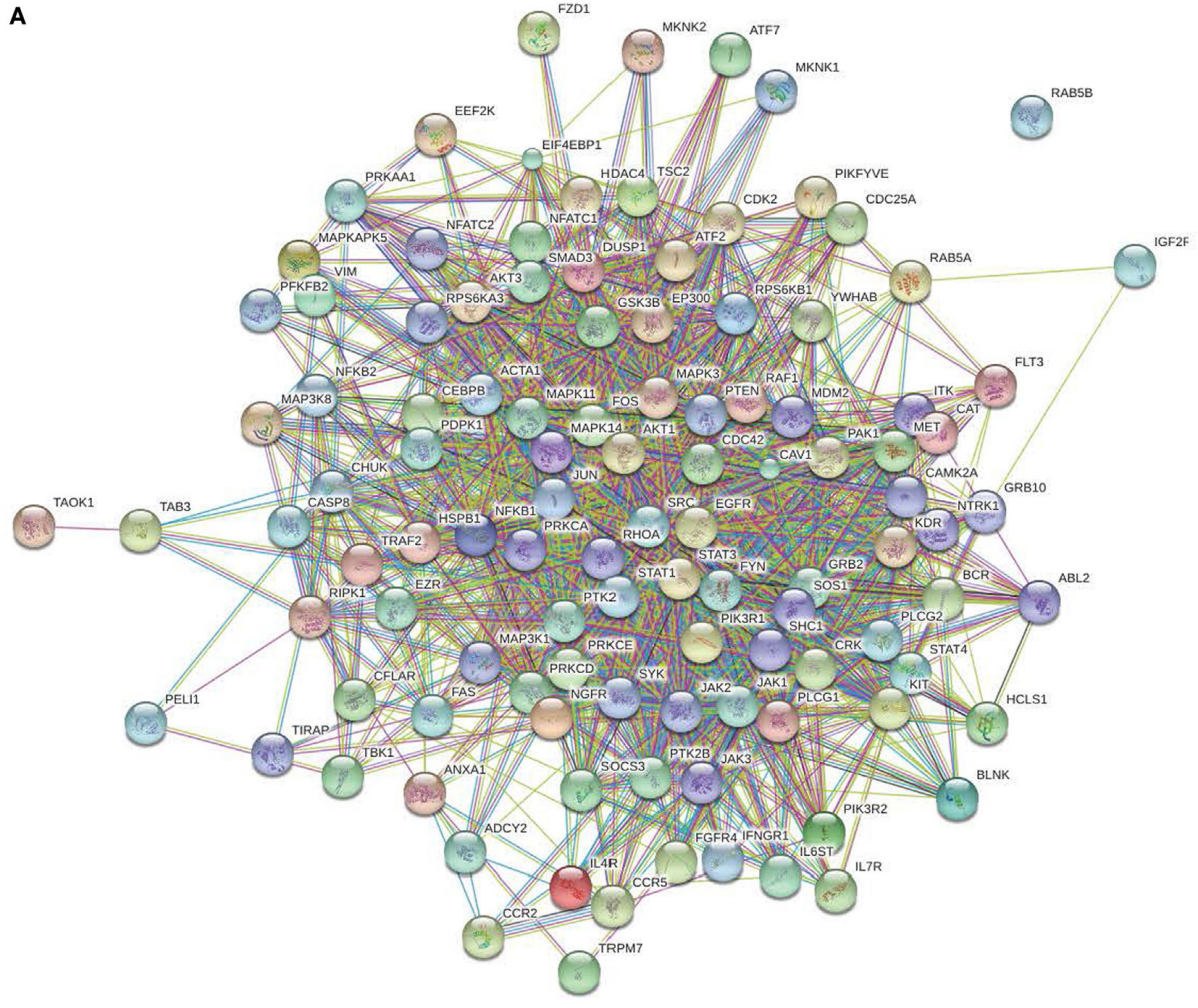

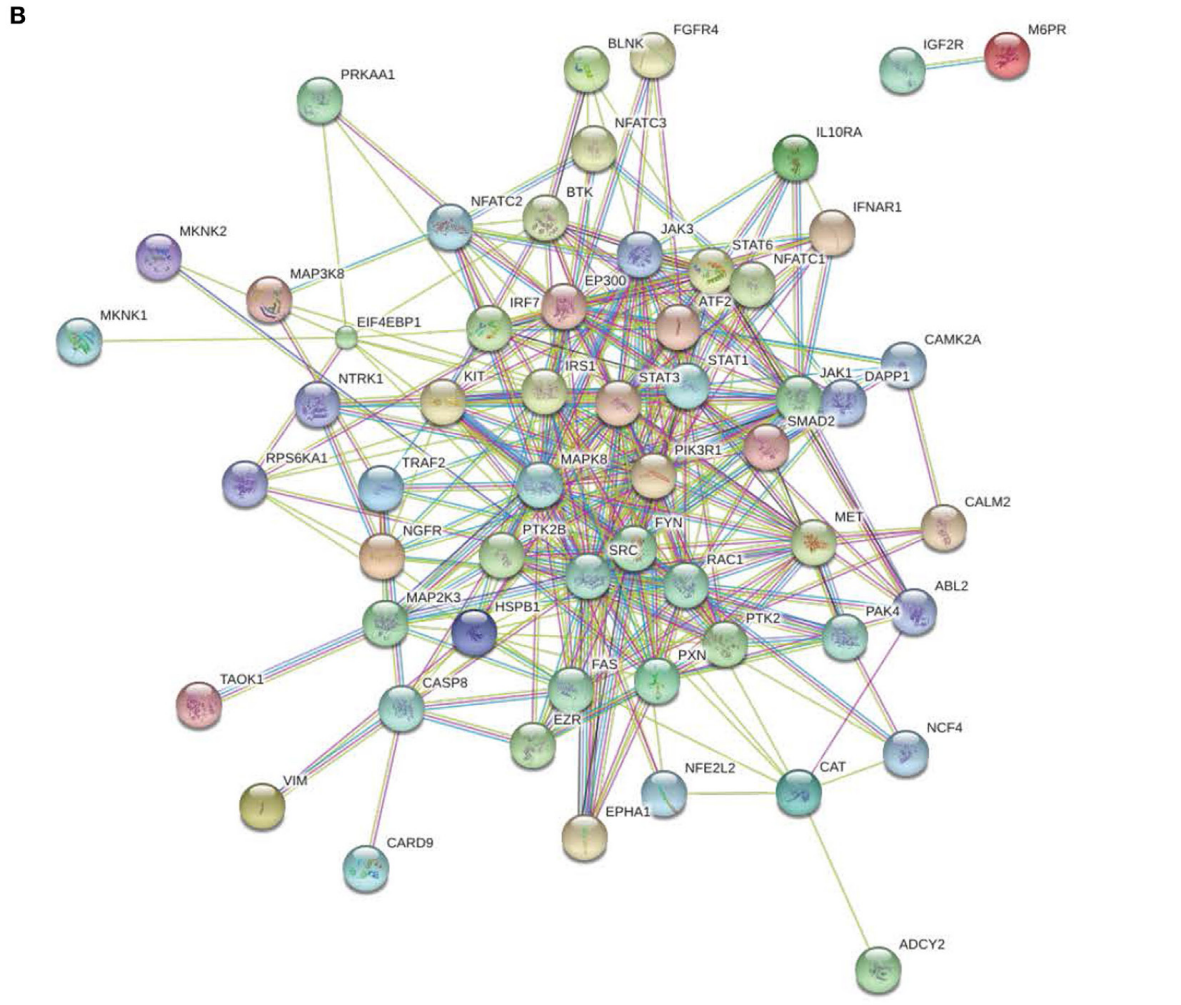

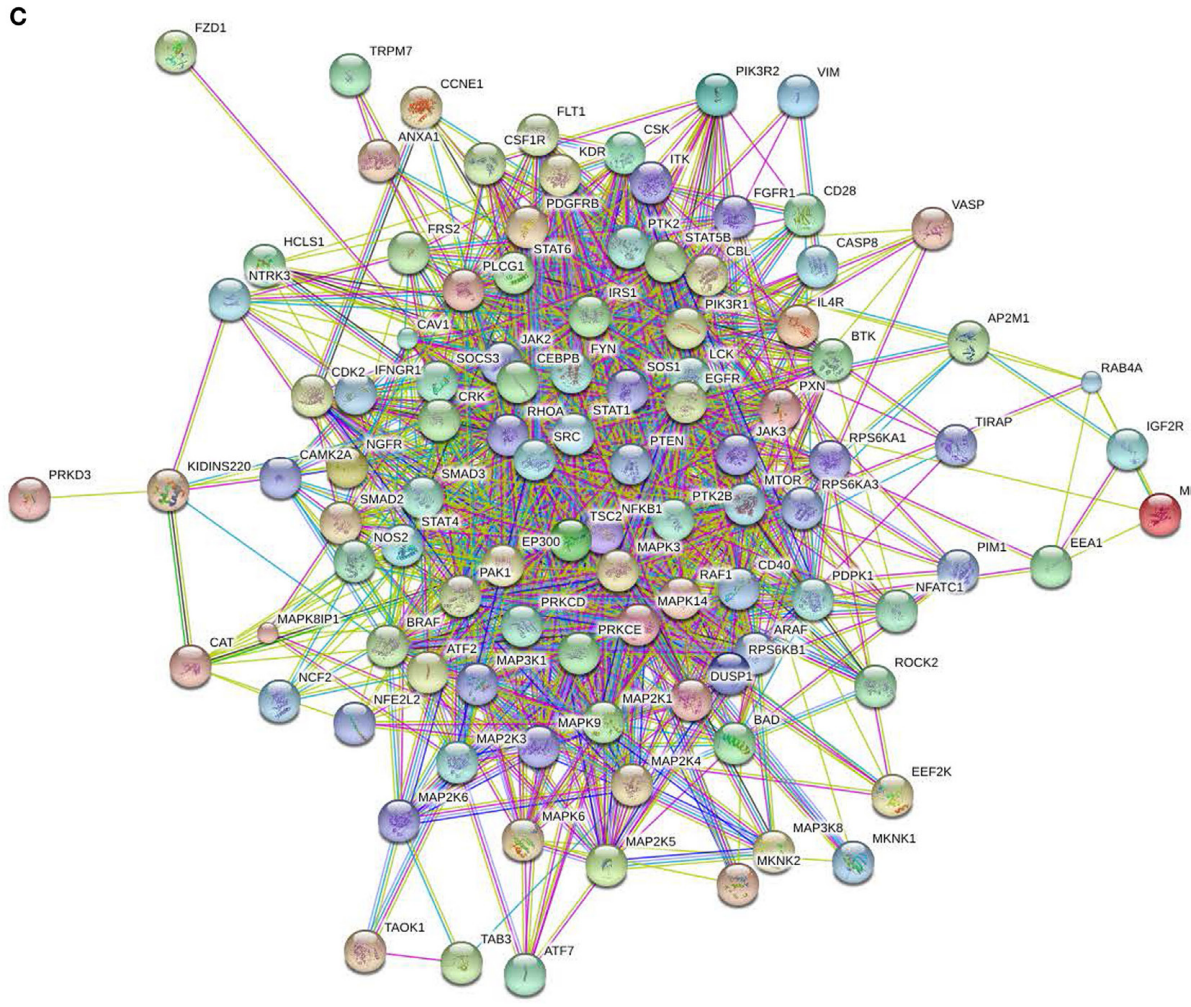

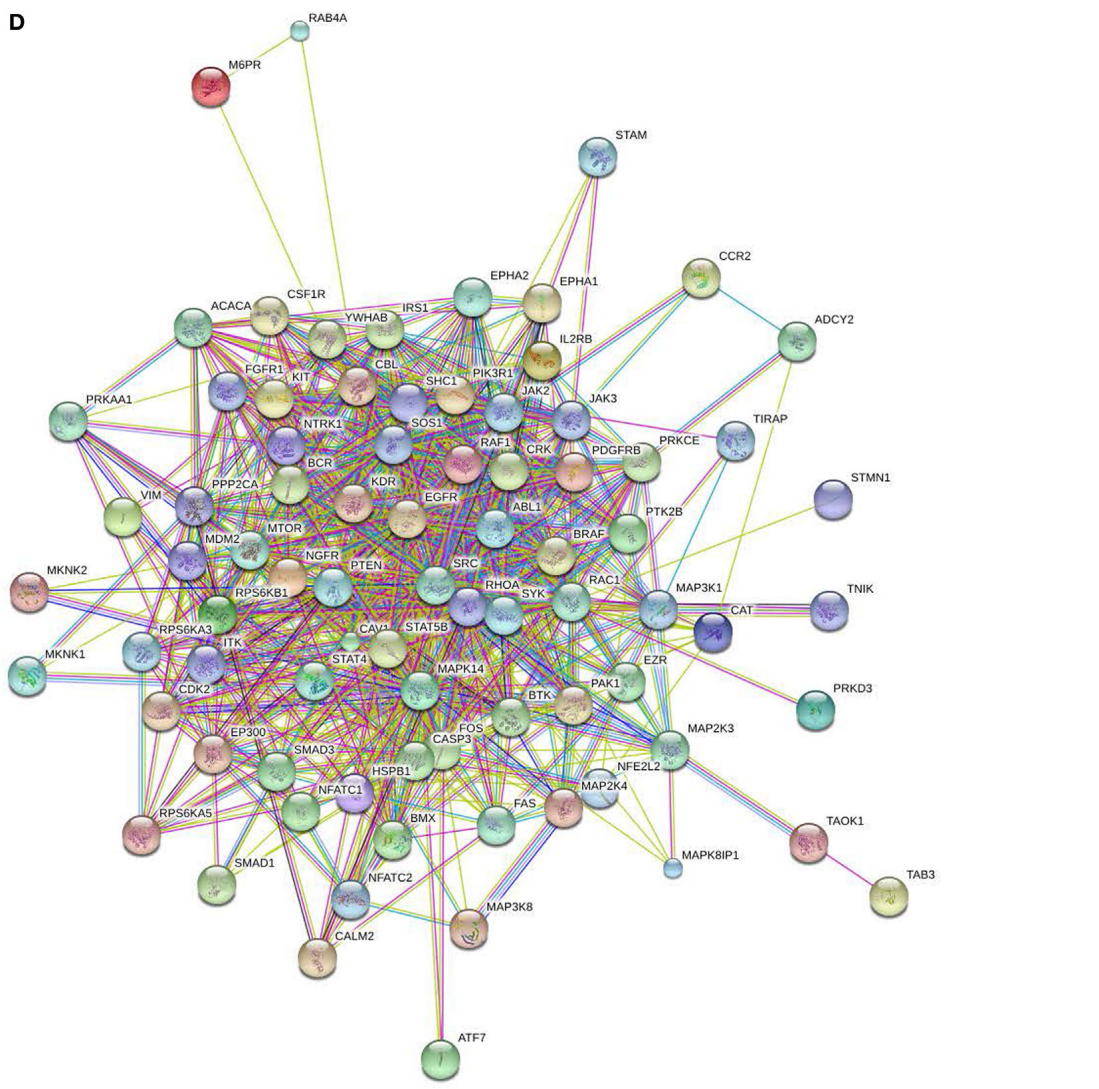

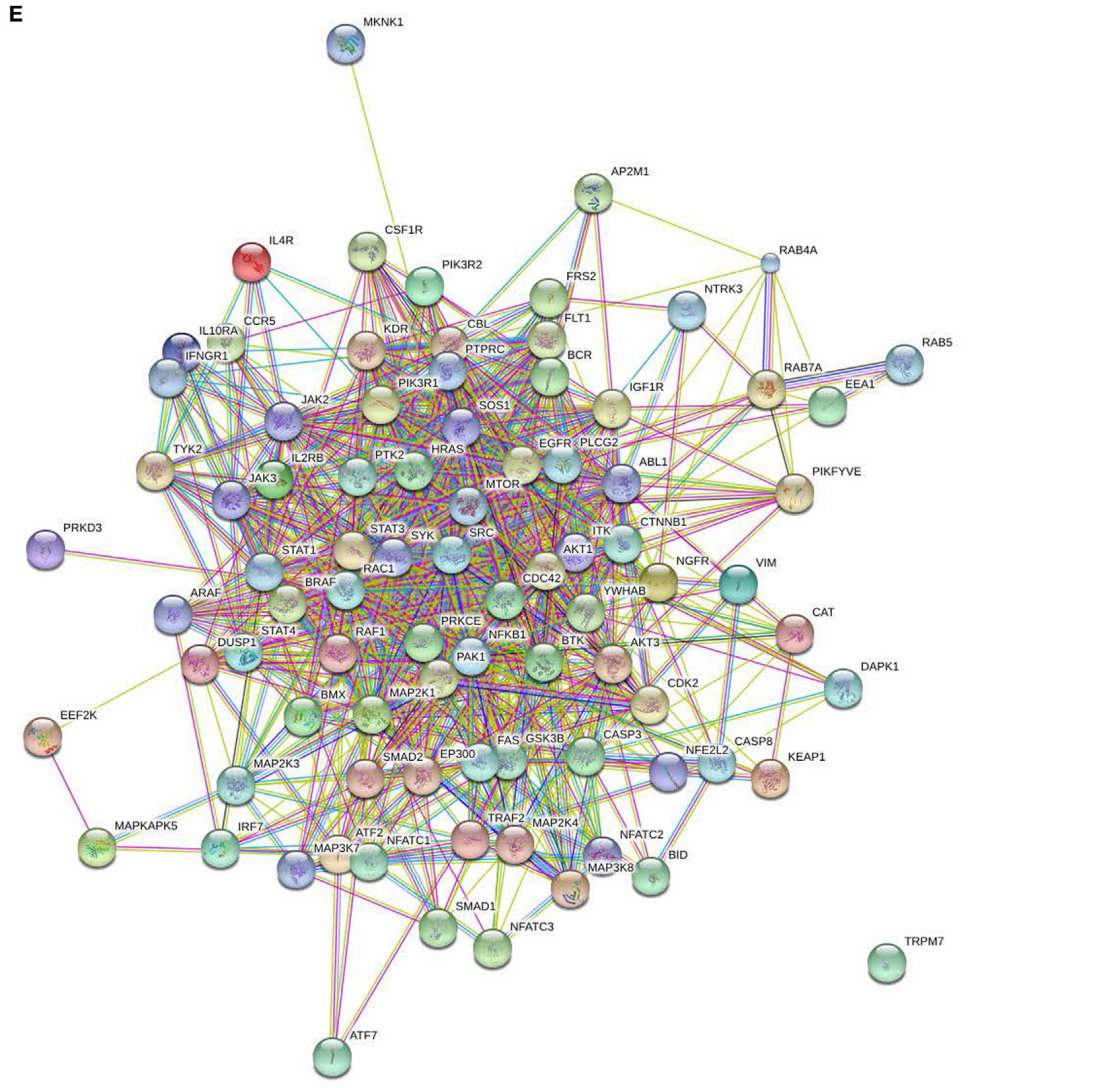

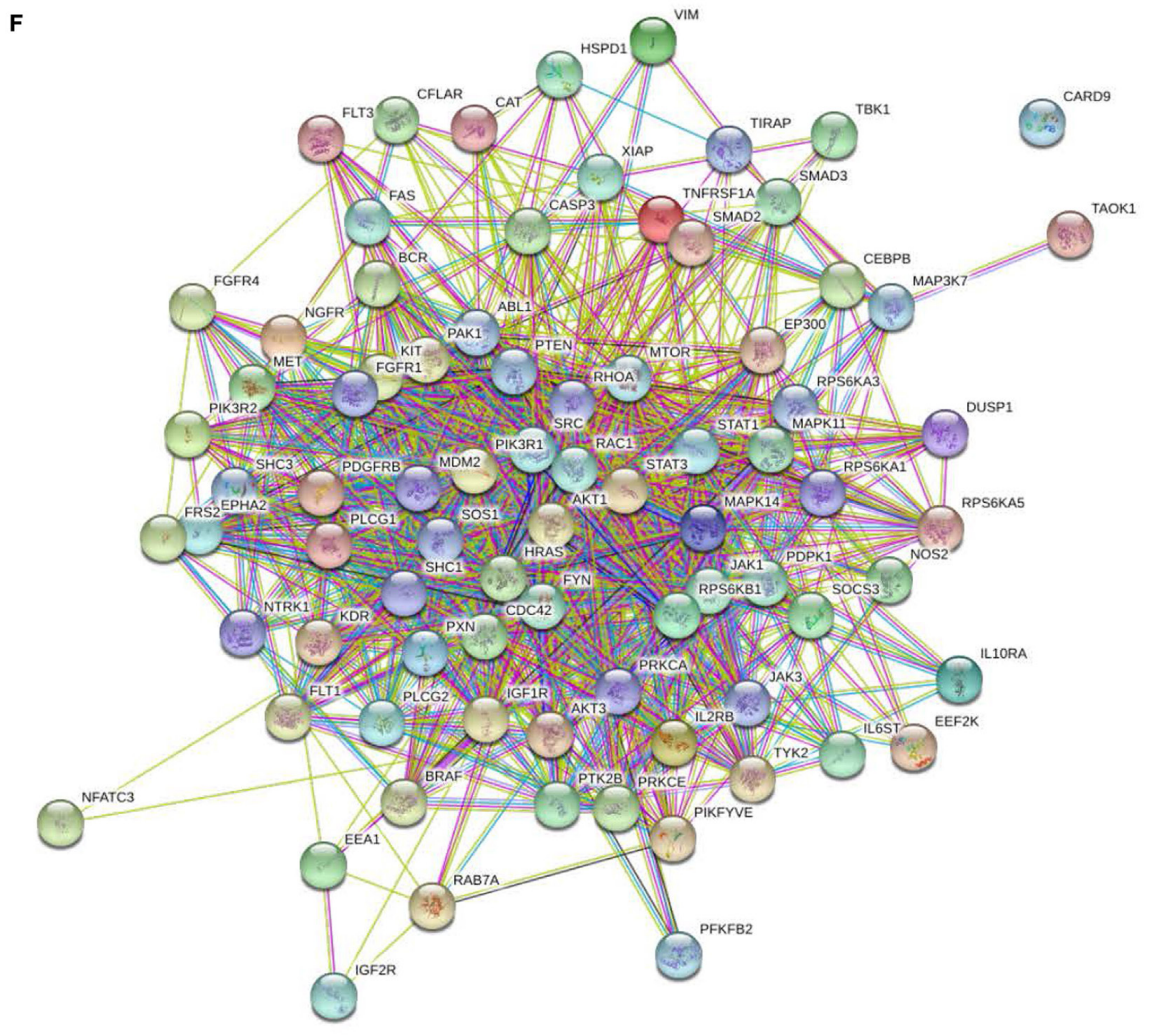
Protein–protein interactions. Predicted protein–protein interactions generated following analysis of significantly different peptides input into the STRING database: **(A)** early low interactions. Protein–protein interactions. Predicted protein–protein interactions generated following analysis of significantly different peptides input into the STRING database: **(B)** early high interactions. Protein–protein interactions. Predicted protein–protein interactions generated following analysis of significantly different peptides input into the STRING database: **(C)** middle low interactions. Protein–protein interactions. Predicted protein–protein interactions generated following analysis of significantly different peptides input into the STRING database: **(D)** middle high interactions. Protein–protein interactions. Predicted protein–protein interactions generated following analysis of significantly different peptides input into the STRING database: **(E)** late low interactions. Protein–protein interactions. Predicted protein–protein interactions generated following analysis of significantly different peptides input into the STRING database: **(F)** late high interactions.

All peptides that were statistically different (*P* ≤ 0.05) for each time were input into the STRING database (22) and diagrams depicting the protein–protein interactions were generated (Figure 2). The most striking difference in the magnitude of the protein–protein interactions was observed between the birds with low or high loads of *S*. Enteritidis colonization at the early time (Figures 2A,B, respectively). The protein–protein interactions for middle low and middle high birds are shown in Figures 2C,D, respectively, and the late low and late high interactions are shown in Figures 2E,F, respectively.

### Validation Using the Antibody Array

To validate the kinome peptide array data, an antibody array containing both pan-specific and phospho-specific antibodies was employed instead of performing individual western blot analyses (8). Analysis of antibody array data *via* the STRING database showed similar GO BP and KEGG pathway activity at the early and middle times as observed with the kinome array; representative samples are shown in Table 5. Though outside the scope of this manuscript, but for validation purposes only, a small number of individual peptides associated with KEGG pathways identified with the kinome array (Tables 2–4) were selected and evaluated to demonstrate consistency between the kinome and antibody arrays. Phospholipase C gamma 1 (PLCG1), protein tyrosine kinase 2 (Pyk2), Raf1, and SMAD2 shared similar phosphorylation/de-phosphorylation patterns and were, in general, in agreement between the two arrays, thus further validating the kinome array results (Table 6). The antibody array was not utilized on the late samples since consistency was demonstrated at the early and middle times. Furthermore, as already described, there were fewer differences between the birds with high and low loads of *S*. Enteritidis at the late time point lessening the applicability of a comparative validation technique.

**Table 5:** Gene ontology (GO) biological process (BP) terms and Kyoto Encyclopedia of Genes and Genomes (KEGG) pathways identified by the antibody array.

BPs		KEGG pathways	
GO ID	Term	High	Low
GO.0050776	Regulation of immune response	Mitogen activated protein kinase (MAPK) signaling pathway	Pathways in cancer
GO.0045087	Innate immune response		MAPK signaling pathway
GO.0002764	Immune response-regulating signaling pathway		Neurotrophin signaling pathway
GO.0002768	Immune response-regulating cell surface receptor signaling pathway		T cell receptor signaling pathway
GO.0002684	Positive regulation of immune system process		Chemokine signaling pathway
GO.0002376	Immune system process		
GO.0048522	Positive regulation of cellular process		
GO.0006952	Defense response		
GO.0007166	Cell surface receptor signaling pathway		

**Table 6:** Fold-change in specific peptides associated with high and low loads of *Salmonella* Enteritidis using the peptide array and validation with the antibody array.

		Early		Middle	
Peptide	Accession no.	Low peptide (Ab)	High peptide (Ab)	Low peptide (Ab)	High peptide (Ab)
Phospholipase C gamma 1	P19174	−1.7^a*^ (−1.8*)	−1.0 (−1.0)	2.4* (ND)	1.2 (1.5*)
Pyk2	Q14289	ND (−1.5*)	1.7* (1.9*)	ND (1.2*)	1.8* (1.3*)
Raf1	P04049	1.5* (ND)	ND (ND)	1.2 (1.7*)	ND (−2.5*)
SMAD2	Q15796	−2.8* (−5.3*)	1.5* (1.9*)	−1.1 (−5.3*)	1.9 (−7.8*)

*(Ab), antibody array; ND, not detected*.

*^a^Fold-change from control*.

*P ≤ 0.05.

### Quantitative Real-time RT-PCR

The expression of IL6 and CXCLi2 mRNA was quantified (40-C*_t_*) in tissue from birds with high and low levels of *S*. Enteritidis cecal colonization at early, middle, and late times. Birds with lower levels of *S*. Enteritidis at the early time point had significantly (*P* ≤ 0.05) higher mRNA expression levels of CXCLi2 than birds with higher loads of *S*. Enteritidis (14.5 and 13.6, respectively). There were no statistical differences in CXCLi2 mRNA expression at the middle and late times. There were no differences in IL6 between birds with high and low loads of *S*. Enteritidis colonization compared to one another or the respective controls at the early, middle, or late times (data not shown).

## Discussion

Laboratory challenges using animal models are a vital component for making scientific advances regardless of the field of study. Despite controlling for host genetics, environmental conditions, and challenge preparation and recovery methodologies, investigators accept there will be a certain amount of variability observed between individual animals. Such variability was observed within the line of birds evaluated in the present study, and despite 100% of the challenged chickens being culture positive for *S*. Enteritidis, the actual numbers of recoverable bacteria varied between individuals (Figure 1). A recent study suggests the differences in bacterial growth and immune responses seen across genetically identical mice is a result of specific immune elements that facilitate the co-regulation and interconnectedness of the innate and adaptive immune responses (23). The observed differences in cecal colonization could also be due, in part, to intermittent shedding. It is widely recognized that chickens shed varying levels of *S*. Enteritidis over time (24, 25). Observing the different levels of cecal colonization in the study presented herein led us to hypothesize that differences may be detectable by evaluating the host kinome response and, therefore, provide valuable insight into the mechanism(s) that either limits or enables *S*. Enteritidis, one of the most important foodborne bacteria, to colonize the chicken ceca.

The results presented herein revealed that birds with high and low levels of cecal colonization with *S*. Enteritidis, at the time of sampling, have distinct kinome profiles (i.e., protein phosphorylation patterns). As such, key BP and immunologically related pathways associated with increased resistance within a single population of birds were identified (Tables 1–4). The signaling pathways that differed between birds with high and low loads of *S*. Enteritidis colonization include those associated with chemokine, Jak–Stat, MAPK, and T cell receptor signaling. Differences in these seminal pathways would be anticipated as several studies in poultry show that strong pro-inflammatory cytokine and chemokine responses are associated with increased resistance against disease (26–30). Moreover, differences within individual components of the MAPK signaling pathway [p38, extracellular signal-regulated kinase (ERK), c-Jun N-terminal kinase (JNK)] have been reported in chickens and turkeys. Genes within the MAPK signaling cascade were mapped and shown to be involved in resistance against *Salmonella* in chickens (31). Another study in chickens showed that increased resistance against *S*. Enteritidis organ invasion is associated with elevated production of p38 and decreased production of JNK (32) while increased production of p38, JNK, and ERK are all influential in determining the level of resistance in turkeys (33). Involvement of the MAPK signaling pathway extends beyond mere immunological responses by the host. In fact, the virulence factors encoded by *Salmonella* can promote either activation or deactivation of the MAPK signaling pathway (34–36), so it is possible the observed changes are a direct result of the bacteria and not necessarily the hosts’ response to the challenge. Further studies are necessary to dissect this complex host–pathogen interaction.

Additionally, KEGG analysis showed that pathways in cancer were significantly different across all times in birds with lower levels of *S*. Enteritidis cecal colonization. The authors are not suggesting the *S*. Enteritidis challenge resulted in cancer in the birds. Of note, studies on the kinome are widely used in cancer research since virtually every cancer displays varying levels of protein and/or lipid kinase dysregulation. Therefore, kinome analysis provides meaningful insight into the pathways and families of kinases involved in specific cancers (37), which would explain why pathways affiliated with cancer are identified in the analysis tools. The specific proteins associated with each of the pathways, including those specifically related to cancer, would also be pivotal in determining the hosts’ immunological response against a challenge and/or disease. As previously shown, kinome analysis is beneficial in dissecting pathways involved in animal studies including bovine viral diarrhea virus (38); *in vitro* responses against toll-like receptor agonists (39), Johne’s disease in cattle (40), and *Salmonella* in chickens (8, 41) demonstrating the technology is useful in providing valuable information into diverse infections that alter the normal host mechanisms.

Changes in cytokine and chemokine expression in chickens are widely reported following *Salmonella enterica* challenges and/or infections. In the current study, mRNA expression of CXCLi2 was upregulated early in the chickens with lower levels of *S*. Enteritidis. This finding is supported by another study showing CXCLi2 is found in the gut of newly hatched chicks and mRNA expression continues to increase the first week post-hatch (42). More specifically, CXCLi2 mRNA is upregulated in *Salmonella-*resistant chickens (27, 43). CXCLi2 (formerly referred to as IL8) is a potent pro-inflammatory chemokine capable of recruiting immune cells, such as heterophils, to the site of infection (44), and heterophil recruitment is associated with increased resistance against *S*. Enteritidis (16). The role of heterophils was not considered in the current study, but increases in CXCLi2 expression have been reported in various cells and tissue types across diverse breeds of chickens (45–47). Therefore, our study is in agreement and indicates increased CXCLi2 is likely a contributing factor to the lower numbers of bacteria seen at the early time. As might be expected, no differences in CXCLi2 were observed at the middle and late times as the infection had become persistent instead of acute (41). In addition to CXCLi2, IL6 mRNA expression has been shown to increase following infection with *S*. Enteritidis (27, 48). No differences were detected in IL6 mRNA expression, but it is possible the timing of sample collection was not optimized to detect this cytokine. Additional studies are required to understand the role of IL6 and CXCLi2 expression and their impact on influencing the load of cecal colonization of *S*. Enteritidis in broilers over a grow out period.

The current study showed that a single line of birds with high and low levels of cecal colonization with *S*. Enteritidis at the time of collection have distinct kinome profiles. These data support the value of peptide arrays and kinome analysis as a powerful molecular tool to identify key mechanisms and pathways that are associated with increased resistance against *S*. Enteritidis cecal colonization in chickens. These findings provide a foundation for future studies to identify the specific markers associated with lower loads of cecal colonization and will focus on the common pathways identified herein, including chemokine, Jak–Stat, MAPK signaling pathways, or pathways in cancer. Identification of specific biomarkers that the poultry industry could use to select individual birds that are more resistant to cecal colonization with *S*. Enteritidis would be beneficial to the industry. This could potentially lead to either fewer *S*. Enteritidis positive birds entering the processing plant or reducing the load of bacteria the birds are carrying and therefore fewer positive chicken products reaching the consumer.

## Ethics Statement

All experiments were conducted according to guidelines established by the USDA animal care and use committee, which operates in accordance with established principles (14). The protocol was approved by the acting USDA Plains Area animal care and use committee that operates at the location where the experiments were carried out.

## Author Contributions

CS was the lead investigator and principal author; MK, HH, KG, CJ, and RA were collaborators and coauthors.

## Conflict of Interest Statement

The authors declare the research was conducted in the absence of any commercial and/or financial relationship that could be construed as a potential conflict of interest.

## Footnotes

^1^www.phosphosite.org.

^2^http://geneontology.org/page/ontology-documentation.

## Abbreviations

BP, biological process; cfu, colony forming units; *C_t_*, threshold cycle; dpi, days post-infection; EDTA, ethylenediaminetetraacetic acid; EGTA, ethylene glycol tetraacetic acid; ERK, extracellular signal-regulated kinase; FDR, false discovery rate; GO, gene ontology; IL, interleukin; JNK, c-Jun N-terminal kinase; KEGG, Kyoto Encyclopedia of Genes and Genomes; MAPK, mitogen-activated protein kinase; PBS, phosphate buffered saline; PIIKA2, Platform for Intelligent Integrated Kinome Analysis; PLCG1, Phospholipase C gamma 1; Pyk2, protein tyrosine kinase 2; qRT-PCR, quantitative real-time Reverse Transcriptase-PCR; *S*. Enteritidis, *Salmonella enterica* serovar Enteritidis; STRING, Search Tool for the Retrieval of Interacting Genes; USDA, United States Department of Agriculture; XLT-NN, XLT4 agar base plates with XLT4 supplement and nalidixic acid and novobiocin.
